# Results comparison of cervical cancer early detection using cerviray ® with VIA test

**DOI:** 10.1186/s13104-025-07086-6

**Published:** 2025-01-22

**Authors:** Ali Budi Harsono, Hadi Susiarno, Dodi Suardi, Kemala Isnainiasih Mantilidewi, Viko Duvadilan Wibowo, Yudi Mulyana Hidayat

**Affiliations:** 1 Department of Obstetrics and Gynecology, Faculty of Medicine, Universitas Padjadjaran–Dr. Hasan Sadikin General Hospital, Bandung, Indonesia; 2 Faculty of Medicine, Universitas Padjadjaran – Dr. Hasan Sadikin General Hospital, Jl. Pasteur No. 38, Bandung, West Java 40161 Indonesia

**Keywords:** Cervical cancer, Cerviray, VIA test, Early detection

## Abstract

**Objectives:**

This study investigates the performance of artificial intelligence (AI) technology, namely Cerviray AI^®^, compared with Cerviray^®^ expert, aiming to compare its sensitivity, specificity, positive predictive value (PPV), and area under the receiver operating characteristic curve (AUC ROC). The Visual Inspection with Acetic Acid (VIA) test is used as the gold standard.

**Results:**

The study involved 44 patients from various health centers in West Java Province. Performance of Cerviray AI^®^, or Cerviray^®^ expert, and lastly VIA tests were compared in their ability to detect pre-cancerous cervical lesions in high-risk women of childbearing age. The current study indicated that Cerviray AI^®^ had a sensitivity of 42.9%, specificity of 100%, PPV of 100%, and ROC AUC values of 71.4%. In comparison, the evaluation of the Cerviray^®^ expert demonstrated a sensitivity of 71.4%, specificity of 97.3%, PPV of 83.3%, and ROC AUC values of 84.4%. In conclusion, the evaluation of Cerviray^®^ expert outperformed Cerviray AI^®^ in ROC AUC values.

**Trial registration:**

Clinical Trials.gov Identifier NCT06518070 Retrospectively registered.

**Supplementary Information:**

The online version contains supplementary material available at 10.1186/s13104-025-07086-6.

## Introduction

Cervical cancer is a cancer caused by Human Papilloma Virus (HPV) infection. Cervical cancer is the eighth most common cancer in the world, with an estimated 662,301 new cases and 348,874 deaths in 2022 worldwide, and the prevalence of HPV infection in women was 5.2%.^1^ About 90% of new cases and deaths from cervical cancer occur in low- and middle-income countries [1]. 

As the causative agent of almost all cases of cervical cancer, HPV can infect both male and female genital regions, including the skin of the vulva, penis, and anus; the lining of the vagina, cervix, and rectum; and the lining of the mouth and throat [2]. Unlike other sexually transmitted infections, most signs and symptoms of HPV are absent. Therefore, most people are unaware of HPV infection in their bodies. HPV types 16 and 18 are the most oncogenic virus types and are responsible for causing more than 75% of cervical cancer cases and most other genital cancers [3]. 

As in our body, many viruses like HPV can cause cancer cells because of its persistent infection. The hypoxic condition of cells and oxidative stress can lead to DNA damage and altered nuclear architecture. This condition becomes a transactivator function in virus-infected cells so the cells can be dormant and become cancerous too [4, 5]. 

Cervical cancer is highly preventable. Nearly all cervical cancer could be prevented by primary prevention with HPV vaccination. The appropriate screening method for secondary prevention to detect precancerous lesions still varies in different countries. In Indonesia, the VIA test is appropriate as a screening method because the VIA test is easy, cheap, accurate, and can be implemented all across the country. The VIA results are immediately available and the treatment can be administered on-site [6]. The VIA test may have drawbacks because it is considered as subjective and operator-dependent, therefore experience and training are needed [7]. WHO recommends the VIA as a screening technique till a low-cost HPV test becomes available in low-income countries [8]. 

Currently, the development of screening for cervical cancer co-testing (the VIA test and HPV DNA test) is still under discussion to become a national program in Indonesia. This co-testing escalates the sensitivity of screening for patients with precancerous lesions or cervical cancer because it can identify the presence of HPV infection before it develops into a precancerous lesion [7]. 

Recent technological advances, including Artificial Intelligence (AI), can improve the quality of care and cost-effectiveness in the medical field [9]. Indonesia is geographically vast, with many small islands causing limited medical access. To assist medical workers in these remote areas, early detection of cervical precancerous lesions may be done by providing handy medical devices. In addition, an integrated AI in the device can support them in deciding the management of suspicious cervical lesions to be referred to or can be observed closely in their health center [7]. 

Cerviray AI^®^ is a technology for diagnosing cervical cancer using a portable colposcope and artificial intelligence-based software (AIDOTNet al.gorithm). This device has 93% sensitivity and 89% specificity).^11^ This product was developed by a company named AIDOT from South Korea, characterized by its use of AI to assist untrained operators in detecting pre-cancerous cervical lesions. It is non-invasive, easy to use, and delivers results within seconds. The tool is particularly useful in areas with limited access to trained professionals for cervical cancer screening. The Cerviray AI^®^ helps make VIA screening more objective. The image of the cervix can be detected by Cerviray AI^®^ as unsatisfactory (inappropriate image for diagnosis), normal, CIN1 (low-grade or mild dysplasia), CIN2/3 (moderate to high-grade dysplasia), and CIN3+ (high-grade dysplasia to invasive cancer). Cerviray AI^®^ provides a telemedicine system that enables remote consultation with experts (Cerviray^®^ expert) [10, 11]. 

Due to the limitations of the VIA test, this Cerviray AI^®^ can be used as a tool for screening in Indonesia while waiting for the implementation of co-testing as a national program as standard screening in this country. This study investigates the performance of Cerviray AI^®^, compared with Cerviray^®^ expert, aiming to compare its sensitivity, specificity, positive predictive value (PPV), and area under the receiver operating characteristic curve (AUC ROC). The VIA test is used as the gold standard. This study uses the VIA test as a comparison to the AI because the VIA test is the most widely used method by healthcare professionals in Indonesia. A study to compare the use of Cerviray AI^®^ to the VIA test has not been conducted in Indonesia before.

## Main text

### Methods

This method uses a non-experimental study design with a cross-sectional approach conducted to compare the performance of Cerviray AI^®^ and Cerviray^®^ expert, using the VIA test as a gold standard.

### Sample size calculation

For samples with small populations, total sampling can be used where the samples included are all those encountered during the research. The number of minimal samples was calculated using the Cochrane formula:

N = z^2^p(1-q)/e^2^.

With this formula, the calculations were done systematically as follows:

*Z*^2^ = (1.96)^2^ = 3.8416.

*p x q* = 0.7 × 0.3 = 0.21.

*e*^2^ = (0,15)^2^ = 0.0225.

Enter the score to the formula:

$$n\, = \,{{3.8416\, \times \,0.21} \over {0.0225}}\, = \,35.8549$$

This calculation obtained results of a minimal sample of 35 patients.

### Cerviray AI^®^

The composition of Cerviray AI^®^ is hardware as a portable colposcope with a camera and LCD touch screen installed. It is also equipped with microSD to save the pictures. The software itself called Cerviray AI^®^ developed by AIDOT Inc. and is compatible with PC also tablets that provide four-stages AI screening results as Normal, CIN 1, CIN2/3, and CIN 3 + according to Bethesda classification. It detects abnormal lesions on the cervix directly (referred as Cerviray AI^®^ ) or it can also be confirmed with expert opinion (referred as Cerviray^®^ expert).

### Subjects

The target population included in this study was high-risk women (sexual workers) around Bandung City. High-risk women were chosen based on the reason that the risk factors for developing cervical cancers are persistent HPV infection, smoking, young age at the first coitus, and multiple sexual partners [12]. One prospective study proved that women with high-risk of persistent HPV infections will have a higher risk of cervical cancer even after 15 years [13]. While the risk of cervical precancerous lesions on the general population is less than 0,15% over 5 years [14]. For that reason, this experiment is conducted as a pilot study to collect more possible women with positive results, thus, screening in high-risk women is appropriate.

The inclusion criterion for this study was high-risk women (sexual workers) in Bandung City who have agreed to participate in the study, while the exclusion criteria for this study was patients who refused to participate in the study.

This study follows the protocol for cervical cancer screening by ASCCP with modification. In our institution, cervical cancer screening uses a VIA test. In this study we compare VIA with Cerviray AI^®^ and Cerviray^®^ expert. All patients with negative results were suggested to repeat screening in 1–3 years. Patients with either VIA (+) or abnormal Cerviray AI^®^ and Cerviray^®^ expert underwent subsequent management, which includes colposcopy. Whenever colposcopy results show normal features, the patients were suggested to repeat screening in one year. If acetowhite lesions were discovered during colposcopy, a colposcopy-directed biopsy was taken, and the histopathology result was used as the gold standard for further treatment.

### Statistical analysis

Data processing was carried out by presenting categorical data in the form of proportion data (%) using tables. Data analysis to determine the accuracy of diagnostic tests comparing VIA with Cerviray AI^®^ or Cerviray^®^ Expert was carried out by analyzing sensitivity, specificity, and positive predictive value (PPV), and Kappa statistics were used to compare consistency between diagnostic tools.

## Results

A total of 44 patients were included in this study. Table 1 shows the results of VIA and Cerviray AI^®^ and Cerviray^®^ expert, as well as the demography characteristics of the patients. Six out of 44 patients (13.6%) were found with either VIA (+) or abnormal Cerviray AI^®^ and Cerviray^®^ expert. The mean age of patients was 28.90± 7.069 with minimum age of 18 years old and a maximum age of 46 years old. The majority of patients were multiparity (19 or 43.2%) and primiparity (17 or 38.6%). The patients were all using contraceptives with 10 patients using pill or oral contraception (20.7%), 14 patients using 3 monthly injections (31.8%), 2 patients using monthly injection (4.5%), and 18 patients using condoms (18%).

**Table 1:** Characteristics of the study population

**Variable**	**Subjects** (*n* = 44)	
VIA(-) or Normal Cerviray AI^®^ and Cerviray^®^ expert VIA(+) or abnormal Cerviray AI^®^ and Cerviray^®^ expert	38 (86.4%) 6 (13.6%)	
Age		
Mean ± SD	28.98±7.254	
Minimum	18	
Maximum	46	
Parity		
Nulliparity	8 (18.2%)	
Primiparity	17 (38.6%)	
Multiparity	19 (43.2%)	
Contraceptive		
Comdoms	18 (40.9%)	
Pill	10 (22.7%)	
Monthly Injection	2 (4.5%)	
3 Monthly Injection	14 (31.8%)	

Supplementary Table 1 presented a colposcopic examination of the six patients that showed Normal/CIN 1. Colposcopy-directed biopsy results of these 6 patients with VIA (+) or abnormal Cerviray^®^ and Cerviray^®^ expert showed the results of CIN I in as many as 3 cases (50%), followed by CIN II in as many as 1 case (16.67%). Malignancy cases in the form of endocervical squamous metaplasia were 1 case (16.67%), and non-malignancy cases were in 1 patient with non-specific chronic inflammation in the cervical region (Supplementary Table 1).

Statistical analysis to determine the accuracy of diagnostic tests comparing VIA with Cerviray AI^®^ or Cerviray^®^ Expert is shown in Table 2. The sensitivity, specificity, PPV, and AUC ROC values of Cerviray AI^®^ were 42.9% (95% CI 12.9–77.3), 100%, 100%, and 71.4% (95% CI 46.2–96.6), respectively. The sensitivity, specificity, PPV, and AUC ROC values of the evaluation of Cerviray^®^ expert were 71.4% (95% CI 35.0-94.6), 97.3% (95% CI 88.6–99.8), 83.3% (95% CI 44.6–99.0), and 84.4% (63.7–100.0), respectively.

**Table 2:** Sensitivity, specificity, PPV, and AUC values of the two diagnostic tools

	Cerviray AI^®^ (*n* = 44)		Cerviray^®^ expert (*n* = 44)	
	%	95% CI	%	95% CI
Sensitivity	42.9	12.9–77.3	71.4	35.0–94.6
Specificity	100	-	97.3	88.6–99.8
PPV	100	-	83.3	44.6–99.0
AUC	71.4	46.2–96.6	84.4	63.7–100.0

PPV: Positive Predictive Value; AUC: Area Under Curve

**Fig. 1:**
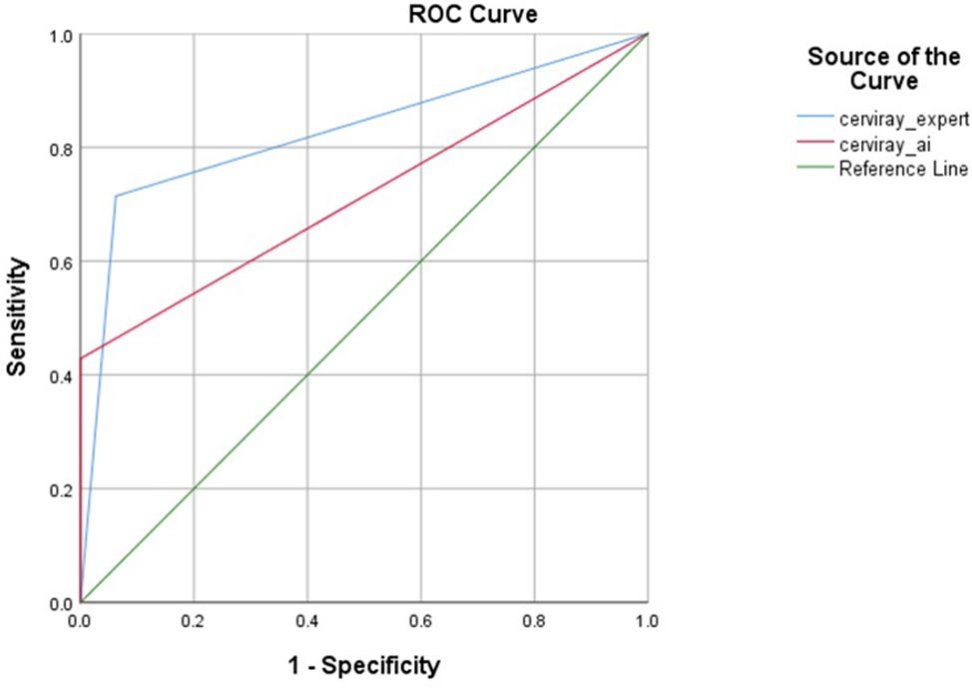
ROC curve of Cerviray AI^®^ and the evaluation of Cerviray^®^ expert against VIA test

The ROC curves showed each tool’s diagnostic ability, where Cerviray^®^ expert showed higher AUC values than Cerviray AI^®^ with AUC values of 84.4% and 71.4%, respectively (Fig. 1). The correlation between the two diagnostic methods was analyzed using Kappa statistics with a value of 0.596 and *p* = 0.02 between Cerviray AI^®^ and Cerviray^®^ expert. This value of Kappa statistics proved that the performance of Cerviray AI^®^ and Cerviray^®^ expert is indeed equivalent and whenever similar results occur between the tools are not accidental.

## Discussion

Cervical cancer is preventable and curable if detected early and managed effectively. However, cervical cancer is the eighth most prevalent cancer worldwide, having claimed the lives of more than 348,874 women in 2022 [15]. 

Screening in the prevention of cervical cancer is a step taken to reduce the incidence of cervical cancer by 50–80%.^2,16^ The VIA method is still the appropriate screening method in Indonesia because it is affordable compared to the HPV DNA test and cytology. However, VIA needs trained health workers to perform the test, and a comprehensive referral follow-up for positive results. One study reported that the VIA test only has a sensitivity of around 66–86% and a specificity of around 66–89%, which suggests variable determinants affect the sensitivity and specificity of this test [16]. 

Some screening tests should be sensitive, easy to obtain, and can be used independently by primary care physicians. Screening tools such as Cerviray^®^ and VIA tests are easy to use and provide information quickly after the examination [10, 17]. 

Cerviray AI^®^ showed sensitivity, specificity, PPV, and ROC AUC values of 42.9%, 100%, 100%, and 71.4%, respectively. The sensitivity of Cerviray AI^®^ is low to assess whether a person has precancerous lesions. However, the 100% PPV value indicates that the tool is reliable when the test is positive. The ROC AUC value of Cerviray AI^®^ was 71.4%, which indicates that Cerviray AI^®^ is still acceptable as a diagnostic tool for precancerous lesions. The result of this study is quite different from the study conducted in the previous study by Kim. In the study by Kim, the Cerviray AI^®^ had sensitivity, specificity, PPV, and ROC AUC values of 74.14%, 83.05%, 81.13%, and 77.7%, respectively. Compared to our study, the study by Kim showed much higher sensitivity value, while our study showed much higher specificity and PPV, yet both are comparable in the ROC AUC values [10]. 

The evaluation of Cerviray^®^ expert results from re-evaluation by experts directly through the Cerviray^®^ website portal. The results of Cerviray^®^ expert in this study showed sensitivity, specificity, PPV, and ROC AUC values of 71.4%, 97.3%, 83.3%, and 84.4%, respectively. The sensitivity of the evaluation of Cerviray^®^ expert is adequate to assess whether a patient has precancerous lesions, supported by sufficient PPV and ROC AUC values. Indeed, Cerviray^®^ expert escalates the Cerviray AI^®^ performance. These results are equivalent to the Kim study, where the evaluation of Cerviray^®^ expert was conducted by two different doctors. The result of Kim study had sensitivity, specificity, PPV, and ROC AUC values from the two doctors are 84.48% and 83.62%, 77.97% and 74.58%, 79.03% and 76.38%, and 79.99% and 76.9% respectively. The difference between this current study and the study in Korea may be due to the different gold standards being used, that is study by Kim used cytology as a reference, whilst our study used the VIA test.

Few diseases can illustrate global inequalities as clearly as cervical cancer. Almost 90% of cervical cancer deaths in 2020 occurred in low- and middle-income countries. This is where the burden of cervical cancer is greatest, as community access to health services is still very limited, and screening and treatment of the disease are not widely implemented [18]. An ambitious, integrated, and inclusive strategy has been developed by WHO to guide the elimination of cervical cancer as a health problem [19]. In the public health sector, AI provides easier decisions for health workers to diagnose. In Indonesia nowadays, we are currently pursuing cervical cancer screening to cover at least a minimal 90% of the population of women of reproductive age as one of the national health programs. We anticipate an increase in the number of women screened for cervical cancer with AI assistance.

Cerviray AI^®^ is a promising applicative innovation in developing cervical cancer screening in resource-limited developing countries, including Indonesia. Cerviray AI^®^ is equipped with AI software and telemedicine features that make VIA screening more objective and less dependent on the experience/competence of the examiner. Cerviray AI^®^ can maintain the advantages of VIA and help overcome its disadvantages.

## Conclusion

This study underscores the potential of Cerviray AI^®^ and the evaluation of Cerviray^®^ expert in cervical cancer diagnosis, highlighting their distinct performance metrics. The findings suggest that the evaluation of Cerviray^®^ expert, enhanced sensitivity and diagnostic accuracy, could serve as a valuable tool in complementing traditional methods.

### Limitations

This study was a single-center study with a limited number of participants, although statistically has already fulfilled the minimal number of samples. The reason this study had difficulties in reaching a large number of participants were there is a hesitation for the patients to undergo early screening for cervical cancer. In addition, this is a preliminary attempt to determine the accuracy of Cerviray AI^®^’s compared to expert assessment of cervical precancerous lesions. Therefore, a larger study with more participants is needed to confirm the findings of this study.

## Electronic supplementary material

Below is the link to the electronic supplementary material.

Supplementary Material 1

## Data Availability

The datasets generated and/or analysed during the current study are not publicly available due to patients’ confidentiality but are available from the corresponding author on reasonable request.

## Abbreviations

AI: Artificial intelligence
PPV: Positive predictive value
AUC ROC: Area under the receiver operating characteristic curve
VIA: Visual inspection with acetic acid
HPV: Human papilloma virus
VIA: Visual inspection with acetic acid
