# Supplementary material for: Results comparison of cervical cancer early detection using cerviray ® with VIA test
Source: BMC Res Notes. 2025 Jan 22;18:30. doi: 10.1186/s13104-025-07086-6 (PMC11752994; doi:10.1186/s13104-025-07086-6)
Supplement: Supplementary file 1 — Supplementary Material 1 [file 13104_2025_7086_MOESM1_ESM.docx]

Supplementary Table 1. Colposcopy and colposcopy-directed biopsy results of patients with VIA (+) or abnormal Cerviray® and Cerviray expert®

| **Colposcopy (based on Swede score)** | **Subjects (n=6)** |
| --- | --- |
| Normal/CIN1 | 6 |
| **Colposcopy-directed biopsy** |  |
| Benign | 1 (16.67%) |
| CIN 1 | 3 (50%) |
| CIN 2-3 | 1 (16.67%) |
| Invasive cancer | 1 (16.67%) |
